# Deep-learning-based ghost imaging

**DOI:** 10.1038/s41598-017-18171-7

**Published:** 2017-12-19

**Authors:** Meng Lyu, Wei Wang, Hao Wang, Haichao Wang, Guowei Li, Ni Chen, Guohai Situ

**Affiliations:** 1 Shanghai Institute of Optics and Fine Mechanics, Chinese Academy of Sciences, Shanghai, 201800 China; 2 University of Chinese Academy of Sciences, Beijing, 100049 China

## Abstract

In this manuscript, we propose a novel framework of computational ghost imaging, i.e., ghost imaging using deep learning (GIDL). With a set of images reconstructed using traditional GI and the corresponding ground-truth counterparts, a deep neural network was trained so that it can learn the sensing model and increase the quality image reconstruction. Moreover, detailed comparisons between the image reconstructed using deep learning and compressive sensing shows that the proposed GIDL has a much better performance in extremely low sampling rate. Numerical simulations and optical experiments were carried out for the demonstration of the proposed GIDL.

## Introduction

Ghost imaging was first demonstrated as a manifest of quantum entanglement^1^ as biphoton source was used. But soon after that it has been demonstrated that the quantum source is not necessary^2^. Despite of the debate on the physics, GI has been demonstrated further by using pseudothermal light generated by dynamically modulating the illumination laser beam with a spatial light modulator (SLM)^3^. Although the source changes, the final image are mostly reconstructed using the correlation of signals from the image arm and the reference arm. The ‘reference’ arm now can be physically unexisted as its function can be calculated with the knoledge of the random phase patterns displayed on the SLM. And thus this technique comes with the term of computational ghost imaging (CGI)^3^. CGI has been used in the study of lensless imaging^4^, X-ray imaging^5,6^ imaging in low light^7^ and and harsh environments^8^. However, the requirement of large number of measurements is one of the main issues that prevent it from practical applications^9–12^. Many efforts have been made to reduce the sampling rate. For example, non-computational^13–15^ and computational methods have been proposed to increase image quality under low sampling rate^9–11,16–20^. In particular, compressive sensing GI (CSGI)^10,16–19^ and iterative GI^11,20^ model the problem of image reconstruction in GI as an optimization problem.

In this letter, we propose a new framework of CGI for high quality image reconstruction under low sampling condition. The proposed method uses deep learning (DL) and thus we term it Ghost imaging using deep learning (GIDL). DL is a machine learning technique for data modelling, and decision making with a neural network trained by a large amount of data^21,22^. The application of machine learning techniques in optical imaging was first proposed by Horisaki *et al*.^23^ who used Support Vector Degression (SVG) architecture to learn the scatterer. In the last two years, we have witnessed the rapid development of the application of deep learning in solving various inverse problems in optical imaging. For example, people have used it in fluorescence lifetime imaging^24^ phase imaging^25,26^ and imaging through scattering media^27,28^ By combining GI and DL, we show in this manuscript that GIDL can also decrease the number of measurements significantly as CSGI, but with much better reconstruction. Also, detailed comparisons between the performances, including the image quality and the noise robustness, of CSGI and GIDL are discussed. Our analysis suggests that the GIDL promises great potentials in applications such as imaging and sensing through harsh environments.

### Numerical Simulation

In ghost imaging, the unknown object, *T*(*x*), is illuminated by a sequence of speckle patterns, *I*_*m*_(*x*), where the subscript integer (*m* = 1…*M*) denotes the *m*^*th*^ illumination. Then, for the *m*^*th*^ speckle, the signal collected by a bucket detector can be written as ${S}_{m}=\int {I}_{m}(x)T(x){\rm{d}}x$. Traditionally, the image reconstructed using GI is obtained by the correlation of the signal fluctuation *δS*_*m*_ with the speckle patterns *δI*_*m*_(*x*)

1 $$O(x)=\langle \delta {S}_{m}\delta {I}_{m}\rangle \mathrm{.}$$

In CGI, the speckle intensities *I*_*m*_(*x*) are calculated numerically from the phase patterns displayed on the SLM.

**Figure 1:**
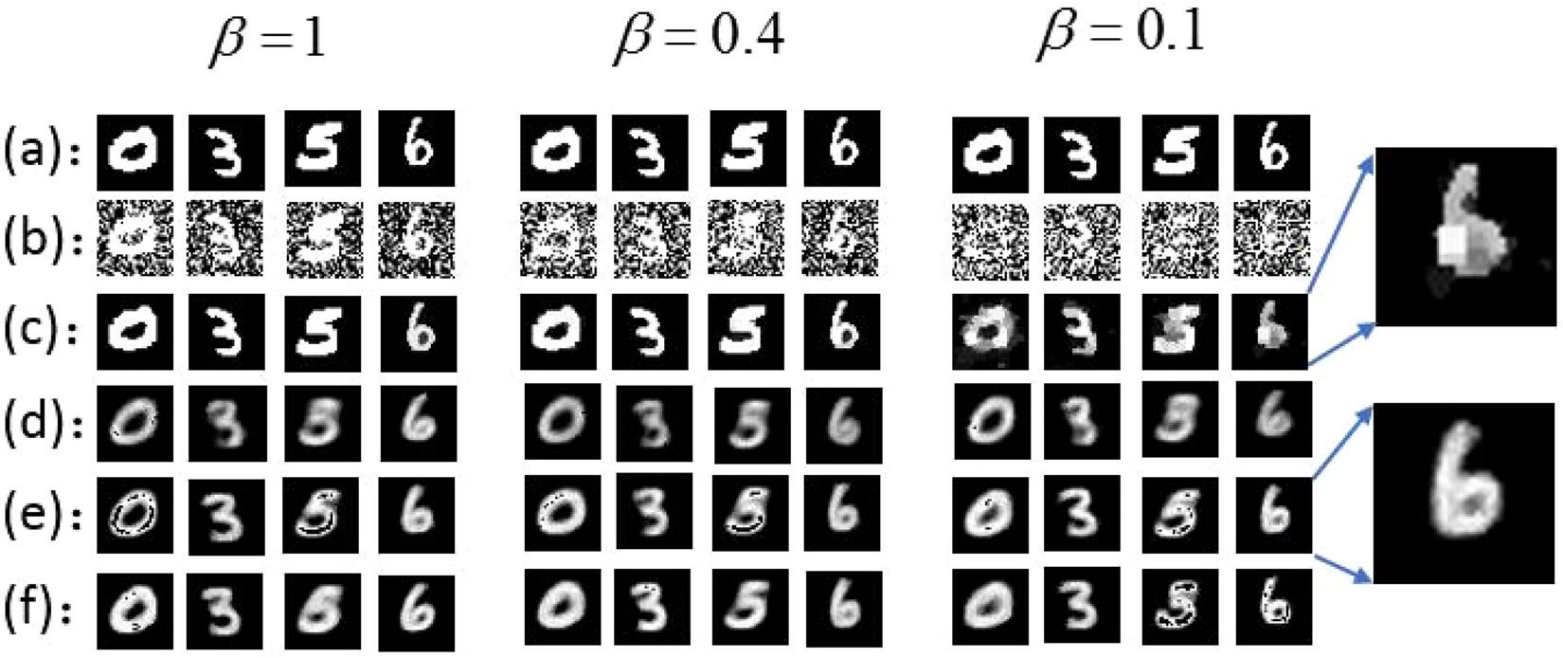
Simulation results of GI, GICS and GIDL. (**a**) Top row: Ground truth objects, (**b**,**c**) Image reconstructed using GI and CSGI for different measurement ratios *β*. (**d**–**f**) Images reconstructed using GIDL (ghost imaging using neural networks) with the number of epochs 10, 100 and 500 respectively. Insets: zoomed in images reconstructed using CSGI and GIDL of the digit ‘6’.

It has been demonstrated that the signal-to-noise ratio (SNR) of the image reconstructed in this way is proportional to the measurement ratio, i.e., the ratio between the number of illumination patterns *M* and the (average) number of speckle in each of these patterns *N*_*spec*_^9,11^, namely, *β* = *M*/*N*_*spec*_. To show how it works, we take the images (digits ‘0’, ‘3’, ‘5’ and ‘6’) shown in Fig. 1(a) as examples in our simulation study. These ground truth images have 32 × 32 pixels. By using the algorithm defined by Eq. (1) one can reconstruct the images as shown in Fig. 1(b). The reconstructed image set in the columns are corresponding to the sampling ratio *β* = 1, 0.4 and 0.1, respectively. The results clearly suggest that, as the ratio *β* decreases from 1 to 0.1, the reconstructed images degrade significantly. The digits can be seen clearly when *β* = 1, although noise appears. But they are completely corrupted by noise when *β* = 0.1.

In order to increase the image quality, one usually sets $\beta \gg 1$ in the the conventional GI and CGI framework, so that the image acquisition procedure is very time-consuming. One popular solution to decrease the acquisition time is to combine GI and compressive sensing (CS) theory^10,16,18^. The CS theory allows the object to be recovered precisely from a smaller number measurements if it is sparse in a presentation domain^29^. So far several frameworks of CSGI have been demonstrated. But high-quality image reconstruction when *β* is small, i.e., $M\ll {N}_{spec}$, is still a challenging problem^10,16,18^. In CSGI, one actually aims at solving the following inverse problem instead of calculating Eq. (1):

2 $$\mathop{{\rm{\min }}}\limits_{T}\parallel \nabla T\parallel 1+\frac{u}{2}\parallel {\bf{A}}T-S{\parallel }_{2}^{2},$$

where ∇*T* is the discrete gradient of *T*, *u* is a weighting factor between the first term and the second term in Eq. (2), which represents the linear model between the image measurement matrix ***A*** and the detected signal vector $S={[{S}_{1},{S}_{2},\ldots ,{S}_{M}]}^{\perp }$, where the symbol ⊥ denotes transposition. In this study, we solve Eq. (2) using the open source CS solver TVAL3^30^ and reconstruct the images. The images reconstructed in this way are shown in Fig. 1(c). Because of the sparse constraint, the measurement ratio *β* for a good reconstruction of the object image can be decreased to 0.1 in our simulation. With a measurement ratio *β* = 0.4, the object can be recovered nearly precisely. However, the image reconstructed using a measurement ratio 0.1 is not so smooth due to the sparse regulation. This problem always exists in CS when the number of measurements is small^29^.

**Figure 2:**
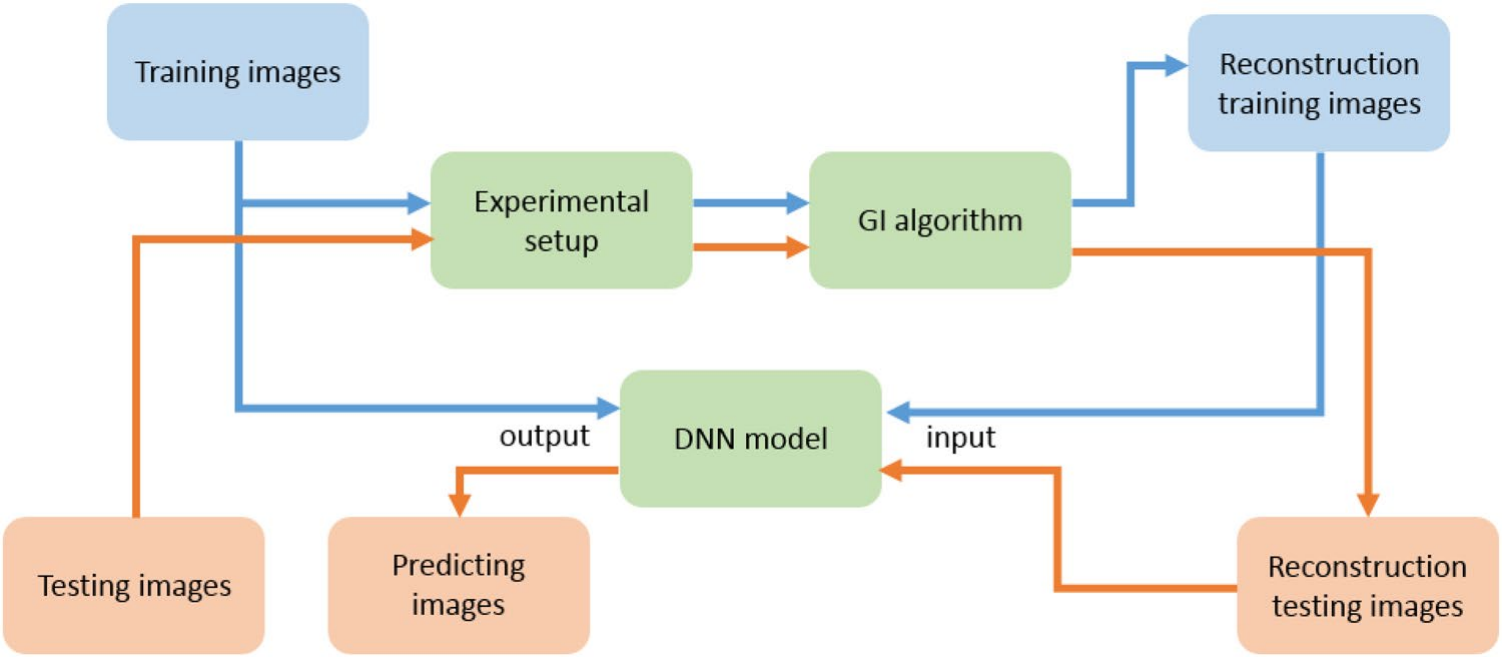
The flowchart of GI using deep neural networks. The blue part represents the training stage and the orange part represents the testing stage.

**Figure 3:**
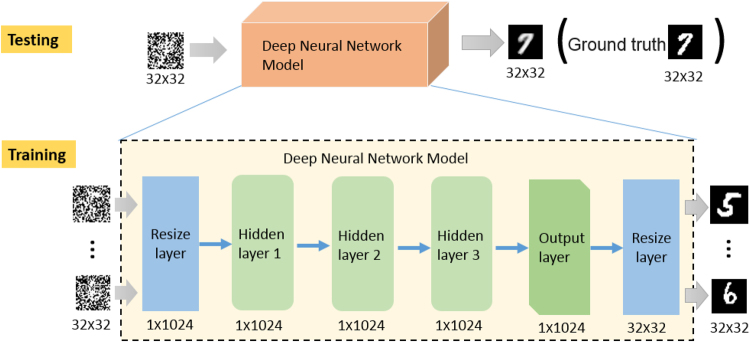
Framework of GI using deep neural networks.

In the proposed scheme, the reconstruction is a two-step process. First, the image is reconstructed from the acquired data directly by solving Eq. (1). As shown in Fig. 1(b), the reconstructed image, *O*, in this way is usually very noisy when *β* is small. But the deep learning is then involved in the second step. The neural network attempts to reconstruct the object image *T* from the noisy, or even, corrupted, *O*. As schematically shown in Figs 2 and 3, the image reconstruction procedures of GIDL is also consist of two steps: training and testing. In the training step, we used a set of 2000 handwritten digits of 32 × 32 pixels in size from the MNIST handwritten digit database^31^ to train the network in our experiments. Some of the digits are shown in Fig. 3. To train the network, we first reconstructed the images of the digits in the training set according to Eq. (1). Then we fed these images together with the corresponding ground-truth digits into the neural network, and optimize the weighting factors that connect every two nerons in two neighboring hidden layers. In this work, we used a deep neural network (DNN) model with two reshaping layers, three hidden layers and one output layer. For demonstration, we used a very simple model. The reshaping layer at the input end shapes the 32 × 32 input speckle pattern into a 1 × 1024 vector. All the hidden layers and the output layer have 1024 neurons. The activate function of these neurons is rectified linear units (ReLU) which allow for faster and effective training of deep neural architectures on large and complex datasets compared with the sigmoid function^32^. The reshaping layer at the output end reshapes the 1 × 1024 vector back to the 32 × 32 image. The loss function and optimization in the DNN model is mean square error (MSE) and stochastic gradient descent (SGD). Once the training is finished (after 500 epochs in our experiments), the DNN can be used to reconstruct the object image *T* from *O*. The program was implemented using Python version 3.5 and the DNN was implemented using Keras framework based on TensorFlow. The GPU-chip NVIDIA Tesla K20c was used to accelerate the computation.

The simulation results plotted in Fig. 1(d–f) show the reconstructed images using GIDL after 10, 100 and 500 training epochs, respectively, for different measurement ratios *β*. From these images we can conclude that: First, as the number of iteration (epoch) increases, the DNN model is better optimized. As a consequence, the reconstructed images becomes clearer and brighter. However, when the number of epoch becomes too large, we observed over-fitting of the data, which yield bit error in the reconstructed images as evidenced by the black spots. Second, the GIDL is not very sensitive to *β*. The MSE values between the images in Fig. 1(f) and the corresponding ground truth images in Fig. 1(a) are all around 0.03 even when *β* = 0.1. This means that by using GIDL for image reconstruction, one can significantly reduce the number of measurements in the GI acquisition procedure. As a consequence, the time efficiency can be improved without sacrifice of image quality. We note that one can achieve the reduction of measurement by using the CSGI framework as well^10^. However, when one takes a closed look at the zoomed-in images of any of the reconstructed digits, say, digit ‘6’, in the inset of Fig. 1, it is clearly seen that the image reconstructed using CSGI is not so smooth because of the regulation, while GIDL gives much better reconstruction. This is one major difference between the images reconstructed using GIDL and CSGI.

An additional advantage of GIDL over other GI frameworks is its robustness against noise. Now we provide a theoretical analysis. For a sufficiently large number of photons, the observed signal *S*_*m*_ by the single pixel camera can be represented by an additive random Gaussian noise^18^

3 $${S}_{m}=\int {I}_{m}(x)T(x){\rm{d}}x+w{\sigma }_{m}{\varepsilon }_{m},$$

where the variance ${w}^{2}{\sigma }_{m}^{2}={w}^{2}\int {I}_{m}(x)T(x){\rm{d}}x$, and *ε*_*m*_ is the standard Gaussian white noise. In the variance, *w* represents the noise level. A larger value of *w* will result in a worse detection image. For speckle field illumination of the same statistics, ${\sigma }_{m}^{2}$ can be regarded as invariant so that one can replace it by a constant value, ${\sigma }^{2}\simeq \Sigma {\sigma }_{m}^{2}/M$.

**Figure 4:**
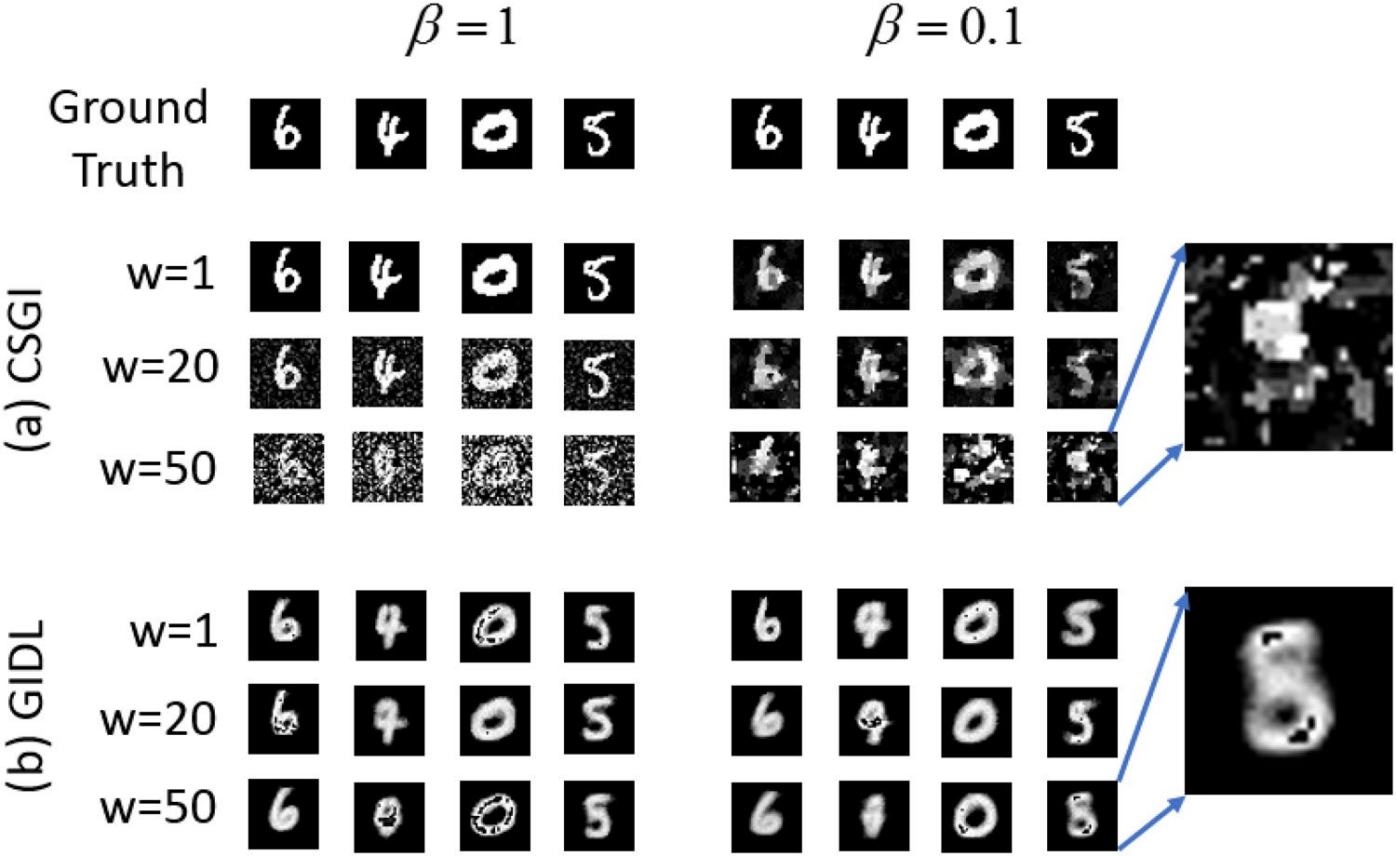
Noise robustness of GIDL. (**a**,**b**) Images reconstructed using CSGI and GIDL under different levels of detection noises. Inset: zoomed in images of the digit object 5 reconstructed using CSGI and GIDL for a high noise level and a low measurement ratio.

The simulation results are shown in Fig. 4. Figure 4(a) shows the images reconstructed using CSGI under different levels of detection noises. For CSGI, when the noise level *w* is small (*w* = 1), the reconstructed images are close to the ground truths, meaning that CSGI can tolerate low level noises. But as the noise increases to a certain level, CSGI fails. The reconstructed image quality is also influenced by the measurement ratio *β* in CSGI. For the case of *w* = 50 and *β* = 0.1, the reconstructed images by CSGI are totally corrupted by noise according to our simulation.

In contrast, GIDL has a much better performance. Figure 4(b) shows the images reconstructed using GIDL under different levels of detection noises. In consist with Fig. 2(d–f), all the images in Fig. 4(b) are smooth as compared to the ones reconstructed using CGSI. The inset shows the zoomed-in images of the digit object ‘5’ reconstructed using CSGI and GIDL for a high noise level and a low measurement ratio (*β* = 0.1). It is seen that the feature details of the digit ‘5’ is clearly recognizable in the image reconstructed by GIDL while it is not by CSGI. This demonstrates the advantage of GIDL over CSGI for imaging and sensing in harsh environments. Although the sparse constraint can be used to decrease the influence of the random detection noise to a certain level, CSGI can not work for high noise levels in which case the linear model Eq. (2) is affected severely. In contrast, in GIDL, the deep learning architecture takes all the noise into account in building up the network model and fits sharply all the partially reconstructed *O* to the corresponding object image *T*. However, when the noise level keep increasing together with the reduction of *β*, the effect of the additive noise cannot be ignored completely. As shown by the digit images ‘4’ and ‘5’ in Fig. 4(b), the reconstructed image becomes blurred, distorting the feature of the object.

### Experiment

**Figure 5:**
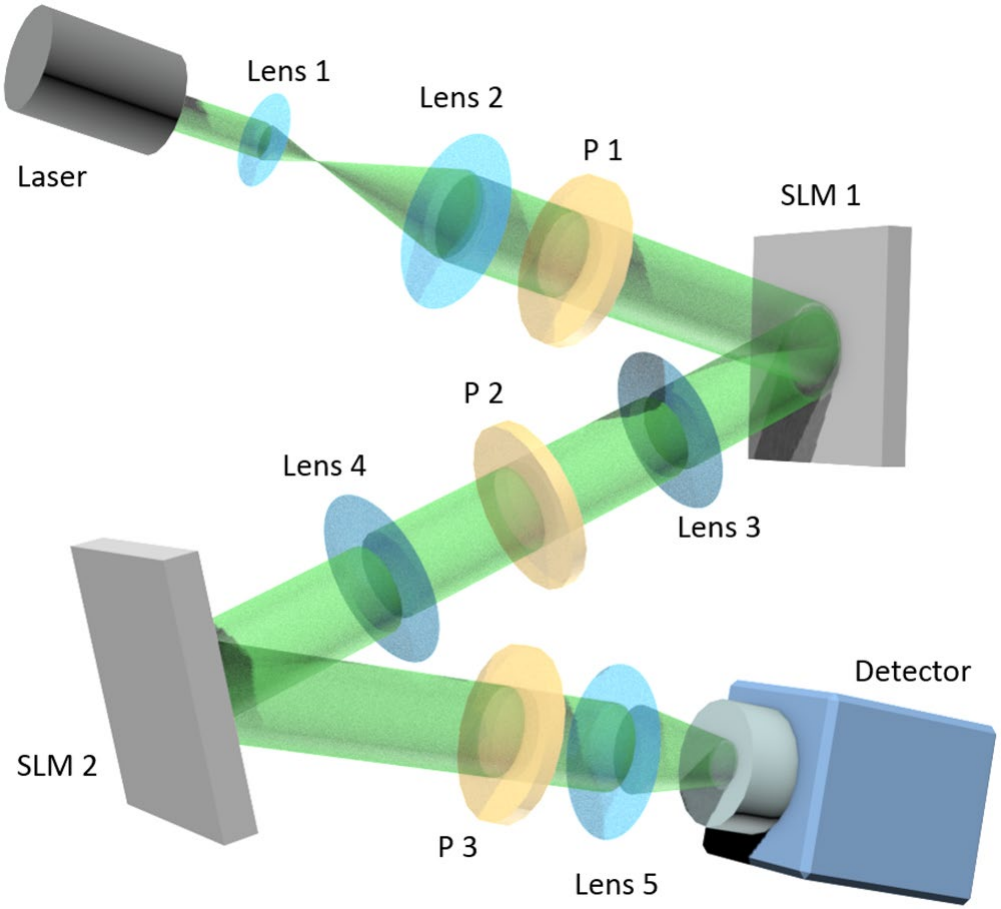
Schematic setup of ghost imaging. P1, P2 and P3 are linear polarizers.

Now we demonstrate the proposed GIDL using some proof-of-principle experiments. We adopted a setup of ghost imaging as the one illustrated in Fig. 5. A laser beam with the wavelength λ = 532 ± 2 nm (Verdi G2 SLM, Coherent, Inc.) was expanded using a 4 f system consisting of lens 1 and lens 2. An SLM 1 (Pluto-Vis, Holoeye Photonics AG) was used to subsequentially display the phase distributions that generate speckle illumination *I*_*m*_, whereas the objects were displayed onto an SLM 2 (Pluto-Vis, Holoeye Photonics AG). The collimated laser beam shone onto SLM 1 and was modulated by the speckle displayed on it. The beam reflected from it was projected onto SLM 2 using the other 4 f system consisting of lens 3 and lens 4. In the setup, P1, P2 and P3 are linear polarizers. P1 and P3 are vertically polarized, and P2 is horizontally polarized, with respect to the laboratory corrdinate, so that to achieve amplitude-only modulation for the SLMs. We displayed different digits from the MNIST database^31^ on SLM 2, serving as the objects in our experiments. The beam reflected from SLM 2 was collected using a sCMOS camera (Zyla 4.2 PLUS sCMOS, Andor Technology Ltd.) because we do not have a bucket detector. We integrated each acquired intensit patterns to produce *S*_*m*_. This does not affect the experimental results except the frame rate and signal amplification because the integration of a recorded intensity pattern acquired by the camera is proportional to the optical power.

**Figure 6:**
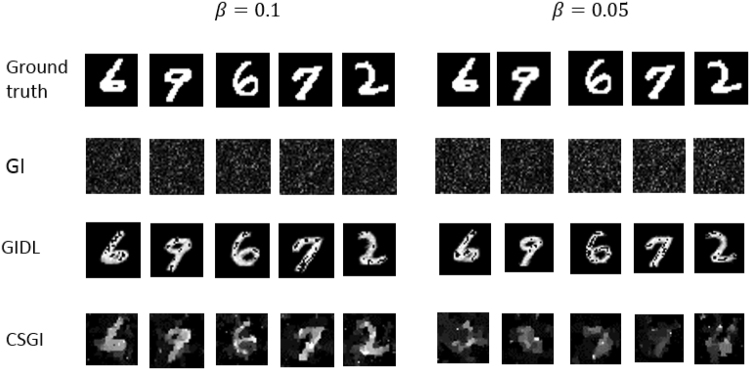
Experimental results under *β* = 0.1 and *β* = 0.05. The images in the first row are the ground truth,the second row shows the images reconstructed using GI, the third row shows the predicted objects using GIDL, and the last row are the images using CSGI.

In the experiments, we used the same training set and testing set as in the simulation. 2000 different digit images in the training set were used to train the network. To demonstrate the proposed scheme, we acquired a very small amount of data to reconstruct the testing digits. In order to speed up the convergence of the DNN model, we used the optimization Adam, and an algorithm for first-order gradient-based optimization of stochastic objective functions^33^, instead of SGD, in the training. The experimental results for *β* = 0.1 and *β* = 0.05 are plotted in Fig. 6. In this figure, the images in the first row are the ground truth images of four digits in the testing set. Due to the small *β* and noise in the system, the reconstructed images using the conventional GI are corrupted by noise as shown in the second row in Fig. 6. One cannot recognize any the visible feature about the target digits from these reconstructed images. However, when we sent them into the trained DNN model, we obtained the corresponding images shown in the third row in Fig. 6. Although they do not resemble exactly the ground truths, the image reconstructed by GIDL contain enough features to recognize. In contrast, the images reconstructed using CSGI are still recognizable at the measurement ratio *β* = 0.1, but become totally corrupted when *β* = 0.05. This suggests that GIDL has a better performance than CSGI at low measurement ratio.

## Conclusion

In conclusion, we have demonstrated the novel technique of GIDL using both numerical and optical experiments. We have analyzed the performance of conventional GI, CSGI and GIDL under different noise and measurement ratio conditions, and observed that GIDL has much better performance than the others especially when the measurement ratio *β* is small. This allows the significant reduction of data acquisition time in ghost imaging, giving a promising solution to this challenges that prohibits GI from practical applications. What’s more, our study opens up new possibility for artificial intellectual techniques in the applications of ghost imaging, and in an even more sense, computational imaging.
